# Left varicocele revealing a nutcracker phenomenon

**DOI:** 10.11604/pamj.2021.39.131.29964

**Published:** 2021-06-15

**Authors:** Guerroum Hind, Benslima Najwa

**Affiliations:** 1 Department of Radiology, Faculty of Medicine, Mohammed VI University of Health Sciences, Cheikh Khalifa International University Hospital, Casablanca, Morocco

**Keywords:** Varicocele, nutcracker phenomenon, Doppler ultrasound, contrast-enhanced, computed tomography

## Image in medicine

A 14-year-old male adolescent, with no notable medical history presented to a medical consultation with pain and swelling in the left testicle of 5 months duration. Physical examination revealed scrotal varicosities, with small testicular volume. Doppler ultrasound (US) of the left pampiniform plexus and the renal vessels confirmed varicocele grade 3, (dilatation of the left pampiniform plexus of veins with reversal flow during the Valsalva maneuver (A,B) and revealed an associated nutcracker phenomenon (C, arrows). Computed tomography (CT) scan with contrast showed also the compression of the left renal vein (RV) between the superior mesenteric artery (SMA), and the abdominal aorta (AO) confirming the nutcracker phenomenon (NCS) (1) (D, arrows). Our patient was treated by inguinal varicocelectomy and had a partial resolution of symptoms after. He was then referred to a nutritionist to increase BMI in order to treat the NCS, before proceeding to invasive methods. Doppler ultrasound of the renal vessels must be performed in case of left varicocele to assess for an associated NCS (2).

**Figure 1:**
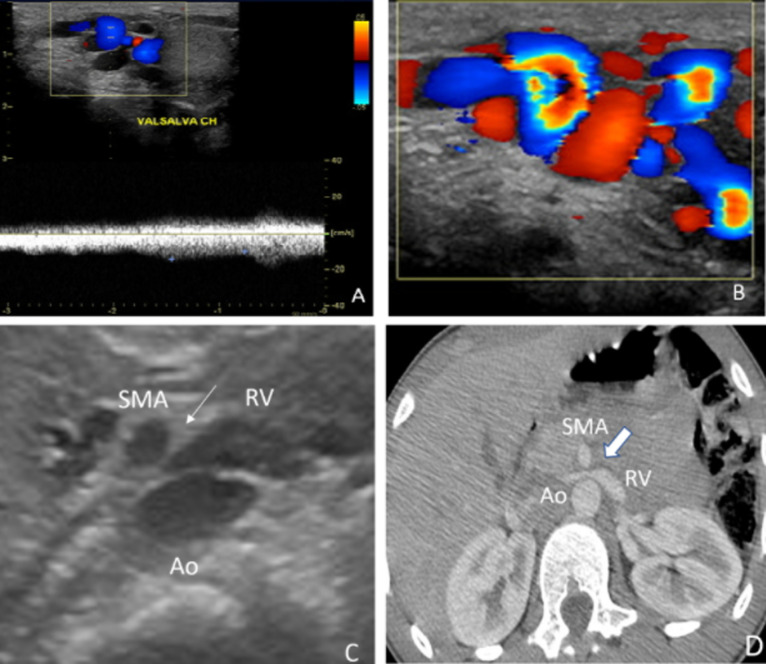
Doppler ultrasound (US) of the left pampiniform plexus and the renal vessels shows varicocele grade 3, as a dilatation of the left pampiniform plexus of veins with reversal flow during the Valsalva maneuver (A, B) and reveals an associated nutcracker phenomenon (C, arrows); computed tomography (CT) scan with contrast shows also the compression of the left renal vein (RV) between the superior mesenteric artery (SMA), and the abdominal aorta (AO) confirming the nutcracker phenomenon (NCS) (D, arrows)

